# Registration and characteristics of clinical trials on traditional Chinese medicine and natural medicines for endometriosis: a comprehensive analysis

**DOI:** 10.3389/fmed.2024.1432815

**Published:** 2024-11-05

**Authors:** Yi Zhao, Yike Wang, Zhu Xue, Yuanyuan Weng, Cencan Xia, Jingyang Lou, Minmin Jiang

**Affiliations:** ^1^Department of Drug Clinical Trials, Women’s Hospital, School of Medicine, Zhejiang University, Hangzhou, Zhejiang, China; ^2^Shulan International Medical College, Zhejiang Shuren University, Hangzhou, Zhejiang, China; ^3^School of Management, Zhejiang Shuren University, Hangzhou, Zhejiang, China; ^4^Beijing Yanchuang Institute of Biomedical Engineering, China Association for Promotion of Health Science and Technology, Beijing, China

**Keywords:** traditional Chinese medicine, natural products, natural medicine, clinical trials, registration, endometriosis

## Abstract

**Objective:**

To investigate the characteristics of clinical trials on traditional Chinese medicine (TCM) or natural medicines for treating endometriosis, aiming to inform future clinical practice and the development of new effective drugs.

**Method:**

The global clinical trial registration platform was searched to identify clinical trials investigating the efficacy of TCM/natural medicine in treating endometriosis. Relevant trials were selected based on stringent inclusion and exclusion criteria. Data entry was performed using Microsoft Excel, while data analysis was conducted using SPSS version 23.

**Results:**

The study encompassed 57 trials, of which ClinicalTrials.gov accounted for 18, ChiCTR for 3, ICRP for 15, and ChiDTR for 21 trials. The number of registrations showed a significant positive correlation with the years. Of the 57 clinical trials, 87.7% were randomized, 63.2% were blinded, 78.9% followed a parallel intervention model, and 56.1% had a sample size below 100. Regarding trial phases, 45.6% of clinical trials did not specify a phase, while Phase 3 and Phase 4 clinical trials accounted for 17.5%. Nine clinical trials involved drugs that are already on the market, including six Chinese patent medicines: Sanjie Zhentong Capsules, Honghua Ruyi Pills, Huayu Sanjie Enema Liquid, Kuntai Capsules, Wenjing Tang, and Xuefu Zhuyu Capsules. Outside China, Iran has the highest number of registrations for natural medicine treatments for endometriosis, with curcumin being the most registered natural medicine.

**Conclusion:**

The analysis reveals that clinical trials on TCM and natural remedies for endometriosis often utilize randomization; however, substantial deficiencies remain in blinding and sample size adequacy. These findings suggest that, despite growing interest in TCM and natural remedies, further methodological improvements are necessary to enhance the credibility of future studies. This research highlights the importance of rigorously designed clinical trials in verifying the safety and efficacy of these alternative therapies, which may influence future therapeutic approaches for managing endometriosis.

## Introduction

Endometriosis is a common chronic gynecological disorder, with an incidence rate of approximately 5 to 15% among women of reproductive age (1, 2). This condition characterized by the presence and proliferation of uterine lining tissue (glands and stroma) outside the uterine cavity, leads to recurrent bleeding, pain, infertility, and the formation of nodules or masses (3, 4). It significantly impacts patients’ quality of life and is a primary cause of female infertility. Currently, traditional treatments for endometriosis primarily consist of drug interventions (both non-hormonal and hormonal) and surgical procedures (which can be conservative or radical) (5). Combined hormonal contraceptives and progestogens are considered the first-line treatment regimen (6). However, long-term use of hormonal therapy can result in various side effects, including headaches, weight gain, and breast pain, as well as potential risks such as osteoporosis (7, 8). Therefore, it is crucial to identify safe and effective alternative therapies.

According to the theory of Traditional Chinese Medicine (TCM), the etiology of endometriosis is attributed to “Blood stasis” (9, 10). TCM boasts a long history of treating endometriosis and continues to be a significant component of modern therapies. Over time, TCM has evolved into a distinctive, personalized, and precise treatment approach that provides promising and unique scientific insights into human health (11, 12). Many renowned TCM gynecologists have developed unique treatment methods and theories based on their extensive clinical experience (13). Moreover, research grounded in Western medical theories has explored the pathological mechanisms underlying TCM treatment of endometriosis, providing preliminary validation of TCM potential mechanisms of action and clinical efficacy (14, 15).

Clinical trials are the standard method for evaluating the efficacy of pharmacological interventions for particular diseases (16, 17) and play a critical role in the development of new drugs (18). Therefore, analyzing registered clinical trial data is essential for guiding clinical practice and advancing future research. Since its launch by the National Institutes of Health (NIH) in 2000, ClinicalTrials.gov has emerged as one of the most significant clinical trial registration platforms globally, accounting for approximately two-thirds of all registered clinical trials (19). Subsequently, various countries have established their own clinical trial registration platforms. In 2005, the China Clinical Trial Registry (ChiCTR) was established and recognized as a primary registration agency by the World Health Organization’s International Clinical Trials Registry Platform (ICTRP) (20). The ICTRP in 2006, integrating 18 international clinical trial registration centers and providing data on over 200,000 clinical trials (21, 22). On September 6, 2013, the China Food and Drug Administration (CFDA) issued Notice No. 28, requiring all drug clinical trials to be registered and publicly disclosed on the “Traditional Chinese Medicine Clinical Trial Registration and Information Disclosure Platform (ChiDTR)” (23). Thus, ClinicalTrials.gov, ChiCTR, ICTRP, and ChiDTR serve as valuable data sources for the clinical registration characteristics of studies on Traditional Chinese Medicine or natural medicines for the treatment of endometriosis worldwide.

Analyzing registered trials for newly developed drugs provides valuable insights into their design, development, and potential shortcomings. In the design and implementation of new drug trials, ethical obligations require consideration of potential risks alongside anticipated benefits (24). To our knowledge, no published papers currently discuss the characteristics of clinical trials on TCM and Natural Medicines for Endometriosis. Furthermore, analyzing registered clinical trials can help identify key drugs and formulations, offering clinicians and researchers important perspectives on TCM and Natural Medicines for Endometriosis. This study aims to analyze the methodological characteristics and key drugs of clinical trials involving TCM and Natural Medicines for Endometriosis registered on ClinicalTrials.gov, ChiCTR, ICTRP, and ChiDTR. The results of this analysis may guide clinical practice and future research directions, particularly in developing new effective therapies.

## Materials and methods

### Search strategy and selection criteria

In the ChiCTR database, the search was conducted using “endometriosis” as the keyword for the “Research Disease Name.” For the ChiDTR database, “Traditional Chinese Medicine/Natural Medicine” was specified under “Drug Type,” with the same indication identified through the keyword “endometriosis” Within the ICRP database, we employed the term “endometriosis” for our search criteria. The search in the clinicaltrials.gov database focused on “endometriosis” under the “condition” field, applying specific filters to exclude trials labeled as “withdrawn,” those including “male” participants, and “patient registries.”

Inclusion criteria included: (1) participants must be adult females; (2) the indication for inclusion was endometriosis; (3) interventions involved the use of natural medicines. Exclusion criteria included: (1) The exclusion criterion was failure to meet any one of the inclusion criteria; (2) incomplete registration, indicated by the platform’s display of missing ethics approval documentation or erroneous uploaded files; (3) studies that were diagnostic, foundational in science, etiological, epidemiological, or survey-based; (4) interventions that utilized synthetic drugs or proprietary Chinese medicines; (5) lack of disclosed specific medications, for example, when the intervention was simply listed as TCM or a combination of Chinese and Western medicine.

This study defines Traditional Chinese Medicine (TCM) or natural medicines used for treating endometriosis as those explicitly stated in the clinical trial protocol, including prescriptions of herbal medicines, herbal extracts, or active ingredients derived through extraction and isolation from herbal medicines.

### Data screening, extraction and analysis

The data screening was executed in four distinct phases. Initially, two researchers independently managed the first three stages; subsequently, they collaboratively reviewed the data during the fourth stage. Disagreements were resolved through consensus or by consultation with a third author. In the first phase, the data, sourced from four clinical trial databases—ClinicalTrials.gov, ChiCTR, ICTRP, and ChiDTR—as delineated in the “Search strategy” section of “Materials and methods,” were imported into an Excel document, with duplicates eliminated based on the clinical trial registration numbers. During the second phase, the data were sifted according to the predefined inclusion and exclusion criteria outlined in the “Materials and methods” section. In the third phase, the removal of redundant trials was carried out by matching the applicant (sponsor) and other IDs across the databases. In the fourth phase, the researchers collaboratively reviewed the data. Disagreements were resolved through consensus or by consultation with a third author. Additionally, this phase entailed cataloging the registration number, study type, registration date, applicant (sponsor), traditional/natural medicine name, and trial design parameters (allocation method, intervention model, blinding, trial phase, and sample size). Data were processed and analyzed using SPSS statistical software version 22.0. Excel 2016 was used for graphing in this study.

## Results

### Search results

**Figure 1:**
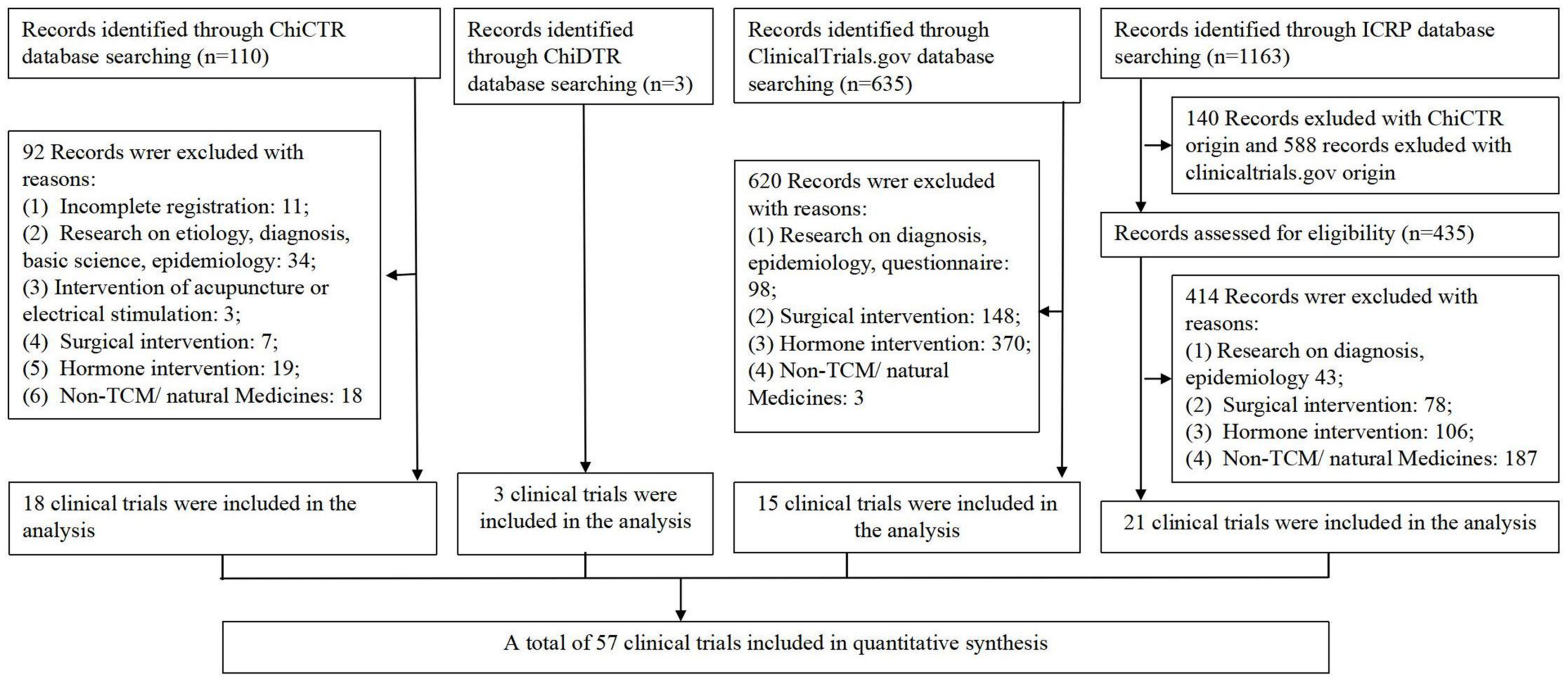
Flow diagram of the study selection process. TCM, Traditional Chinese Medicine; ChiCTR, Chinese Clinical Trial Register; ICTRP, Chinese Drug Clinical Trial Registration and Information Publicity Platform (ChiDTRInternational Clinical Trials Registry Platform).

The detailed retrieval process was shown in flow diagram (Figure 1). Following the aforementioned retrieval method, this study obtained 110, 3, 635, and 1,163 results from the ChiCTR, ChiDTR, ClinicalTrials.gov, and ICTRP platforms, respectively. As the ICTRP platform encompasses clinical trials registered on ClinicalTrials.gov and ChiCTR, trials duplicating these records were excluded during the selection phase. In compliance with the established inclusion and exclusion criteria, the ClinicalTrials.gov, ChiCTR, ICTRP, and ChiDTR platforms yielded 18, 3, 15, and 21 clinical trials concerning herbal/natural medicine treatments for endometriosis, respectively, resulting in a total of 57 trials included in this study.

### General characteristics of the included clinical trials

**Figure 2:**
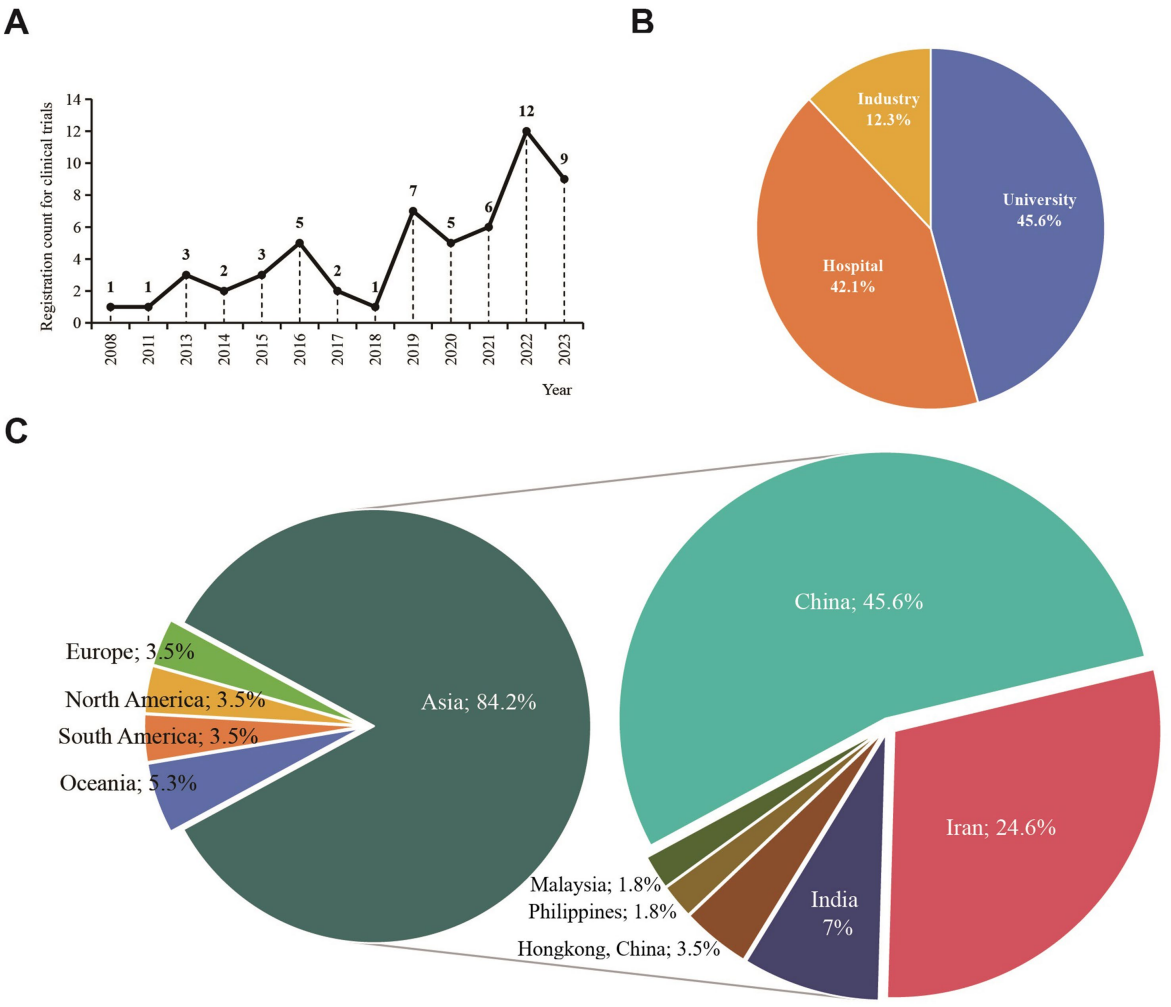
General characteristics of the included clinical trials encompassing TCM/natural medicine as interventions for endometriosis. (A) the relationship of time and number of registration; (B) Distribution of applicant institutions among different industries; (C) Distribution of applicant institutions’ region.

Of the 57 clinical trials encompassed by the research, the greatest number were registered in 2022, accounting for 12 trials, with 2023 trailing at 9 trials. Figure 2A elaborates on the registration details across different years. Linear regression analysis indicated significant differences between the years and the number of registrations. The registration count for clinical trials addressing endometriosis with TCM/natural remedies has markedly risen in recent years (*F* = 17.088, *p* = 0.002). The applicant institutions for these clinical trials were divided among universities (45.6%), hospitals (42.1%), and industry (12.3%), with specific proportions shown in Figure 2B. The majority of the applying institutions were from Asia (84.2%), with China having the highest proportion (45.6%), followed by Iran (24.6%), as shown in the distribution in Figure 2C.

### Design characteristics of the included clinical trials

**Figure 3:**
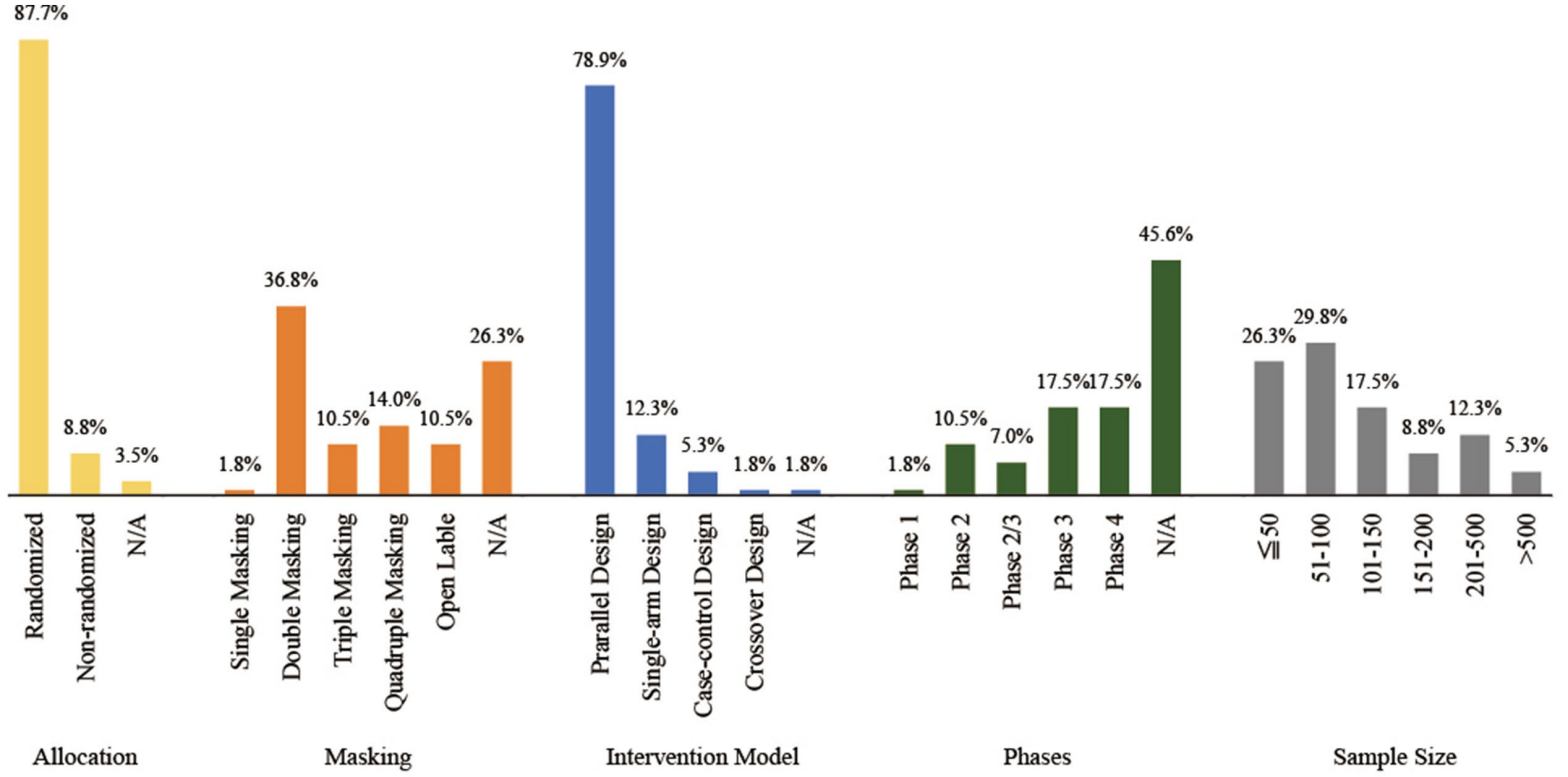
Design characteristics of the included clinical trials. N/A represent not available.

Of the 57 clinical trials included, the majority (87.7%) employed a randomized design, 63.2% of the trials used blinding, with 36.8% adopting a double-blind design, and 78.9% chose a parallel design as the intervention model. Regarding the phases of clinical trials, 45.6% did not specify the phase, while Phase 3 and Phase 4 clinical trials constituted 17.5% of the total. As for sample size, more than half (56.1%) of the clinical trials had fewer than 100 participants. Based on the above, we believe that clinical trials for herbal/natural medicine treatments for endometriosis should increasingly adopt blinding designs and expand sample sizes, in order to achieve higher quality clinical research and to increase their credibility. For detailed characteristics of the clinical trial designs, please refer to Figure 3.

### TCM or natural medicine was used to treat endometriosis in the included clinical trials

A total of 26 clinical trials, focusing on the application of TCM/natural medicine for the treatment of endometriosis, have been registered across the aforementioned four platforms. Notably, all of these trials were conducted exclusively by applicants from China. It is worth mentioning that 50% (13/26) of these trials failed to specify the clinical stage, with the exception of a single trial registered on the ChiCTR platform. Of these 26 clinical trials, 18 (69.2%) trials were from ChiCTR, 3 (11.6%) were from ChiDTR, and 5 (19.2%) were from Clinicaltrials.gov. This indicates a preference among Chinese researchers to register with ChiCTR. The main reason for this preference is that ChiDTR is primarily a clinical trial registration platform for marketing purposes, requiring strict approval by the CFDA. On the other hand, Clinicaltrials.gov is a clinical trial registration platform in the United States. Among the 26 trials, 9 of them involve drugs that have already been approved and put on the market. These drugs include 6 TCM: Sanjie Zhentong Capsules, Honghua Ruyi Pills, Huayu Sanjie Enema Liquid, Kuntai Capsules, Wenjing Tang, and Xuefu Zhuyu Capsules (refer to Table 1 for more details).

**Table 1:** The clinical trials conducted by Chinese applicants on the use of TCM/natural medicines for treating endometriosis.

Trial ID	Database	Phase	Drug name (Chinese name)	Marketed^*^
ChiCTR2300070047	ChiCTR	N/A	Neiyifang Decoction	No
ChiCTR2300069698	ChiCTR	N/A	Yiqi Xiaozheng Granules	No
ChiCTR2300069429	ChiCTR	N/A	Gengnian Zishen Oral Liquid	No
ChiCTR2200066946	ChiCTR	N/A	Guiqiong Xiaoyi Fang	No
ChiCTR2200066925	ChiCTR	N/A	Guiqiong Xiaoyi Fang	No
ChiCTR2200058583	ChiCTR	N/A	Wenjing Tang	Yes^#^
ChiCTR2200057987	ChiCTR	Phase 1	Zishen Quyu Jiedu Granules	No
ChiCTR2200056830	ChiCTR	N/A	Neiyifang Granules	No
ChiCTR2100045119	ChiCTR	N/A	Xiaoliu Fang	No
ChiCTR2100042830	ChiCTR	N/A	Yangjing Zhongyu Tang	No
ChiCTR2000040639	ChiCTR	N/A	Yangjing Zhongyu Tang	No
ChiCTR2000036735	ChiCTR	N/A	Gegensu Tablets	No
ChiCTR1900028624	ChiCTR	Phase 4	Kuntai Capsules	Yes
ChiCTR1900027665	ChiCTR	N/A	Yishen Xiaozheng Granules	No
ChiCTR1900027189	ChiCTR	Phase 4	Sanjie Zhentong Capsules	Yes
ChiCTR-IPR-17013692	ChiCTR	Phase 4	Sanjie Zhentong Capsules	Yes
ChiCTR-IPC-16008214	ChiCTR	Phase 4	Huayu Sanjie Enema Liquid	Yes
ChiCTR-TRC-13003283	ChiCTR	Phase 4	Sanjie Zhentong Capsules	Yes
CTR20140292	ChiDTR	Phase 2	Zhidan Huayu Capsules	No
CTR20132622	ChiDTR	Phase 3	Neiyi Tongjing Granule	No
CTR20140107	ChiDTR	N/A	Zhenggong Capsules	No
NCT04218487	Clinicaltrials.gov	Phase 4	Xuefu Zhuyu Capsules	Yes
NCT02676713	Clinicaltrials.gov	Phase 2	Fufang Zhongcaoyao	No
NCT02031523	Clinicaltrials.gov	Phase 4	Sanjie Zhentong Capsules	Yes
NCT04942015	Clinicaltrials.gov	Phase 4	Honghua Ruyi Pills	Yes
NCT02832271	Clinicaltrials.gov	Phase 2	Epigallocatechin Gallate	No

N/A represent not available; ^#^marketed as Wenjing pills; *Data from the official website of the Chinese State Food and Drug Administration (https://www.nmpa.gov.cn/datasearch/home-index.html).

Apart from China, Iran has the highest number of registrations of natural medicines for the treatment of endometriosis, with a total of 14 related clinical trials. Among these trials, curcumin is the most commonly registered natural drug (refer to Table 2 for more information).

**Table 2:** The clinical trials conducted by Iran applicants on the use of natural medicines for treating endometriosis.

Trial ID	Phase	Drug name	Plant source
IRCT20220115053713N4	Phase 2/3	Ziziphus jujube, Ginseng, and Punica	*Ziziphus abyssinica* Hochst. ex A.Rich.; *Ginseng quinquefolium* (L.) Alph.Wood; *Punica granatum* L.
IRCT20200925048836N4	Phase 3	*Silybum marianum*	*Silybum eburneum* Coss. & Durieu
IRCT20221111056469N1	N/A	Curcumin	*Curcuma aeruginosa* Roxb.
IRCT20220408054455N1	Phase 3	Achillea Cretica	*Achillea cretica* L.
IRCT20170923036334N4	N/A	Curcumin	*Curcuma aeruginosa* Roxb.
IRCT20191207045636N1	Phase 2	*Cymbopogon citratus* oil	*Cymbopogon citratus* (DC.) Stapf
IRCT20201121049457N1	Phase 3	Curcumin	*Curcuma aeruginosa* Roxb.
IRCT20190625044004N1	Phase 3	Chamomile and flaxseed oil	*Matricaria recutita* L.
IRCT20120718010324N66	Phase 3	Curcumin	*Curcuma aeruginosa* Roxb.
IRCT20200701047981N1	Phase 3	Ayurveda	Fennel root and seeds, chicory root and seeds, celery seed, cucumber seed, badrang cucumber seeds, melon seed, vinegar, Water, red sugar
IRCT2015101724569N1	Phase 2	Resveratrol	*Polygonum cuspidatum* Siebold & Zucc. or *Vitis acerifolia* Raf.
IRCT201501059463N34	N/A	Garlic tablets	*Allium sativum* L.
IRCT201501059463N35	N/A	Garlic tablets	*Allium sativum* L.
NCT05983224	N/A	Quercetin	Various food sources like apples, berries, cabbage, and onions

N/A represent not available.

## Discussion

This study found that most applicants for clinical trials on TCM and natural medicines for endometriosis are primarily from institutions in China and Iran. While this geographic concentration is significant, it must be viewed in light of the region’s rich cultural heritage. The prevalence of these studies may be heavily influenced by longstanding cultural traditions, rather than solely by robust scientific evidence. This is particularly important when considering the historical significance of traditional medicine in Asia, which includes systems like TCM, Ayurveda, and Iranian Traditional Medicine (25). These systems, rooted in natural medicinal substances and herbal remedies, have developed over millennia (26). The longevity and evolution of these practices, such as TCM’s origins in ancient China and its continuous development over thousands of years, highlight the profound impact of cultural factors on their use (27). Therefore, interpreting the geographical distribution of trials requires caution, as cultural influences likely play a significant role in shaping the research landscape.

This investigation identified merely three clinical trials focused on the commercialization of TCM for endometriosis treatment, all of which have yet to receive market approval. This implies that in the span of nearly 10 years since 2013, no new pharmaceutical products of TCM for treating endometriosis have been granted market approval. The primary reason may be that the complex composition of TCM, shaped by factors such as production region, climatic conditions, and harvesting periods, poses significant challenges to quality control (28, 29), which has contributed to the historically low number of registered clinical trials for new TCMs (30). However, advancements in modern science and the refinement of China’s pharmaceutical regulatory policies have partially alleviated these challenges. Consequently, since 2020, there has been a notable increase in registration applications for new TCMs, accompanied by a substantial rise in clinical trial registrations (31). These findings correspond with the upward trend observed in this study, indicating that the number of registered clinical trials focused on treating endometriosis with TCM/natural remedies has significantly increased, particularly after 2019.

The defining characteristics of high-quality clinical trials include randomization, blinding, parallel structuring, and the selection of an appropriate sample size (32–34). The results of this study reveal that although most clinical trials on TCM and natural medicines for endometriosis utilize a randomized design, some trials still lack key methodological components, such as a control group, randomization, or blinding. Among the trials analyzed, a significant 36.8% were not blinded, which could potentially compromise the objectivity of the results. Blinding is a crucial criterion for high-quality clinical trials, as it ensures that findings remain free from the subjective preferences or biases of participants, investigators, or assessors (35). A detailed review of the ChiCTR platform suggests that placing the blinding option separately from the main design features, at the end, may cause researchers to easily overlook it during trial registration. This survey also revealed that over half of the clinical trials on Traditional Chinese Medicine and natural medicines for endometriosis enrolled fewer than 100 participants, and the majority of the trial protocols omitted the method for estimating sample sizes. A sample size that is too small, coupled with the absence of strict sample size estimates, can lead to the generation of false negative results, hindering the detection of true differences between different interventions (36). Therefore, future clinical trials should enhance their quality and confidence by adopting reasonable sample size estimation, implementing randomized control, and employing blinded design.

Of the clinical trials included in this research, drugs involved in nine trials are commercially available, including six Chinese patent medicines: Sanjie Zhentong Capsules, Honghua Ruyi Pills, Huayu Sanjie Enema Liquid, Kuntai Capsules, Wenjing Tang, and Xuefu Zhuyu Capsules. Four of these TCM—Sanjie Zhentong Capsules, Huayu Sanjie Enema Liquid, Kuntai Capsules, and Xuefu Zhuyu Capsules—are included in the “Integrated Chinese and Western Medicine Clinical Guidelines for endometriosis” issued by the Gynecology Committee of the Chinese Association of Integrative Medicine (10). This further indicates that the results of high-quality clinical trials have been widely recognized by professionals. The Honghua Ruyi Pill is a refined Tibetan medicine derived from the classical formula “Twenty-Five Flavor Gelsemium Pills” (37). Wenjing Tang is derived from the Southern Song dynasty’s esteemed medical practitioner Chen Ziming’s collection “Fu-Ren Da Quan Liang Fang (38, 39).”

TCM and natural therapies for endometriosis have emerged as a research hotspot, with studies showing their potential to alleviate symptoms such as dysmenorrhea and reduce adnexal masses (40). However, several key issues persist that require further investigation to establish the effectiveness and safety of these treatments. The underlying mechanisms by which TCM and natural therapies exert their effects, particularly when combined with hormonal or pain-relief medications, are not yet fully elucidated. This underscores the need for future research to carefully evaluate the safety and efficacy of such combinations. Furthermore, existing studies are generally small in scale, which highlights the need for larger, high-quality clinical trials designed with rigorous methodologies to confirm the therapeutic potential of TCM and natural therapies for endometriosis (40, 41). Additionally, the potential side effects and long-term safety of these treatments, especially in combination with conventional therapies, have not been adequately investigated. To strengthen the evidence base, future studies should focus on multicenter, randomized controlled trials with blinding, along with long-term follow-up studies to assess sustained outcomes and safety profiles. Furthermore, collaboration between researchers in Western medicine and TCM may facilitate the development of integrative treatment models and enhance the overall understanding of endometriosis management.

This study has inherent limitations that necessitate cautious interpretation of its findings. While our analysis included four major clinical trial registration platforms—ClinicalTrials.gov, ChiCTR, ICTRP, and ChiDTR—we recognize that it is impossible to encompass all registries, particularly those within a single country, such as China’s two primary platforms: ChiCTR, recognized as a principal registry by ICTRP, and ChiDTR, established by the CFDA for new drug applications. The ICTRP’s periodic updates of data from 18 international clinical trial registries may experience temporal delays, and trials registered by non-English-speaking researchers could introduce biases during language translation. Furthermore, cultural differences in medical practices, including the significant role of traditional medicine in Asia, and the differential regulation of herbal remedies—classified as drugs in Asia but as food or dietary supplements in Western countries—may affect the interpretation of our findings. Additionally, only a small proportion of the clinical trials included in this study have published their results on clinical trial registration platforms, limiting further investigation into the efficacy and adverse reactions of these trials.

## Conclusion

This study analyzed 57 clinical trials on TCM and natural remedies for endometriosis. These trials, predominantly conducted in China and Iran, often reflect cultural practices as much as scientific rigor. Despite the frequent use of randomization, many trials lack key methodological elements, such as blinding and adequate sample sizes, which seriously undermines their reliability. The study underscores the necessity for more rigorously designed and larger-scale trials to thoroughly evaluate the efficacy and safety of these treatments, particularly when used in conjunction with conventional therapies.
